# Somatostatin Receptor Expression and Theranostic Applications Across Non-Neuroendocrine Tumors: A Molecular-to-Clinical Review

**DOI:** 10.3390/ijms27198687

**Published:** 2026-09-28

**Authors:** Shaobo Li, Justine Maes, Alex Maes, Sylvie Rottey, Christophe Van de Wiele

**Affiliations:** 1 Department of Diagnostic Sciences, Ghent University, 9000 Ghent, Belgium; 2 Department of Fundamental and Applied Medical Sciences, Ghent University, 9000 Ghent, Belgium; 3 Department of Nuclear Medicine, AZ Groeninge, 8500 Kortrijk, Belgium; 4 Department of Medical Imaging and Pathology, KU Leuven, 3000 Leuven, Belgium; 5 Department of Medical Oncology, Ghent University Hospital, 9000 Ghent, Belgium; 6 Cancer Research Institute Ghent, 9000 Ghent, Belgium

**Keywords:** somatostatin receptor, non-neuroendocrine tumors, molecular imaging, tumor microenvironment, peptide receptor radionuclide therapy

## Abstract

Somatostatin receptor (SSTR)–targeted imaging and therapy are established approaches for neuroendocrine tumors, but accumulating evidence indicates that SSTRs are also expressed in various non-neuroendocrine tumors. This review aimed to summarize their expression patterns, imaging characteristics, and theranostic potential. Published evidence was reviewed for central nervous system tumors, head and neck cancers, thyroid carcinoma, lung cancer, breast cancer, lymphoma, and melanoma, with particular attention to the cellular localization of SSTR expression and the mechanisms underlying radiotracer uptake. Meningioma, non-keratinizing nasopharyngeal carcinoma, and selected estrogen receptor–positive breast cancers show relatively prominent tumor-cell SSTR expression. In several other malignancies, SSTR expression may predominantly involve tumor-associated macrophages, endothelial cells, fibroblasts, or other stromal components. Imaging findings are heterogeneous across and within tumor types. Preliminary studies suggest that peptide receptor radionuclide therapy may provide clinical benefit in selected patients with SSTR-positive non-neuroendocrine tumors, although the available evidence remains limited and largely non-prospective. Further studies integrating quantitative molecular imaging, spatial pathology, and molecular profiling are needed to clarify receptor biology, establish patient-selection criteria, and define the clinical role of SSTR-targeted theranostics beyond neuroendocrine tumors.

## 1. Introduction

Somatostatin receptors (SSTRs), as members of the G protein-coupled receptor family, have long been a cornerstone in the management of neuroendocrine tumors (NETs), where their overexpression enables targeted diagnostic imaging and peptide receptor radionuclide therapy (PRRT) [1]. Radioligands such as [^68^Ga]Ga-DOTATATE for positron emission tomography (PET) and [^177^Lu]Lu-DOTATATE for therapy have profoundly transformed patient outcomes by enabling precise lesion detection and delivering durable responses in advanced disease. However, emerging molecular and imaging studies reveal that SSTR expression extends well beyond NETs, manifesting across a spectrum of non-neuroendocrine tumors (non-NETs). This expression often originates from neoplastic cells themselves, peritumoral vasculature, tumor-associated macrophages (TAMs), or cancer-associated fibroblasts (CAFs) within the tumor microenvironment, thereby expanding the therapeutic horizons of precision oncology [2,3,4]. Among the five SSTR subtypes, SSTR2A is the predominant isoform expressed in human tissues and the principal binding target of clinically used radiolabeled somatostatin analogues. The coexistence of epithelial, vascular, and immune–stromal sources of SSTR2A expression provides the biological basis for the heterogeneous imaging patterns observed across non-NETs and explains why tumors lacking tumor-cell SSTR expression may still demonstrate significant radiotracer uptake.

The heterogeneity of SSTR expression in non-NETs is both striking and clinically valuable. For example, meningiomas demonstrate robust SSTR2 positivity in 79–100% of cases, supporting the superior efficacy of [^68^Ga]Ga-DOTATATE PET/CT for postoperative recurrence detection and margin delineation [5,6,7]. In contrast, in gliomas, tracer uptake is likely influenced by a combination of blood–brain barrier disruption and, in some cases, low-level receptor-mediated binding, although their relative contributions remain to be fully elucidated [8]. Among head and neck malignancies, non-keratinizing nasopharyngeal carcinoma (NPC)—closely associated with Epstein–Barr virus (EBV) infection—displays SSTR2 overexpression in over 80% of EBV-positive tumors, positioning it as a promising biomarker to enhance the specificity of [^18^F]F-FDG PET/CT [9]. In estrogen receptor-positive (ER+) breast cancer, SSTR2 expression is heterogeneous, with some studies reporting positivity rates that may support molecular imaging for hypometabolic metastases that elude FDG detection [10,11]. Even in melanoma, the cellular origin of SSTR-targeted tracer uptake remains uncertain, and a stromal or immune-cell contribution has not yet been confirmed by tissue-localization studies [12].

These insights challenge established SSTR paradigms and highlight their theranostic potential in non-NETs. Early single-photon emission computed tomography (SPECT) agents, such as [^111^In]In-pentetreotide, have been validated for meningioma surveillance and solitary pulmonary nodule characterization, while next-generation ^18^F-labeled tracers (e.g., [^18^F]F-SiTATE) offer enhanced contrast.

To prepare this review, we performed a literature search of PubMed, Embase, Web of Science, and Scopus for publications dated from 1 January 2015 to 31 December 2025, using combinations of the terms “somatostatin receptor”, “SSTR2”, “non-neuroendocrine tumors”, “SSTR imaging”, “DOTATATE”, “DOTATOC”, and “peptide receptor radionuclide therapy”. Reference lists of key articles were screened to identify additional eligible studies, including those published before 2015. The final database search was completed in December 2025. Both clinical and preclinical studies were considered, including molecular analyses, immunohistochemistry, SSTR-targeted SPECT and PET imaging, and theranostic investigations. This review focused on SSTR expression, imaging findings, and therapeutic relevance in selected non-NET tumor types, with particular emphasis on tumor types for which clinical imaging or treatment data were available. Molecular and preclinical findings were included to provide biological context. Because of heterogeneity in study design and endpoints, evidence was synthesized narratively without formal meta-analysis.

## 2. Results

### 2.1. Central Nervous System Tumors

#### 2.1.1. Meningioma

Meningiomas are the most prevalent primary intracranial neoplasms in adults, accounting for nearly 40% of all central nervous system tumors [13]. Patients with aggressive meningiomas that recur after surgery or radiotherapy have limited effective treatment options and a poor prognosis [14]. It is reported that SSTR expression can be detected in meningioma cells. Dutour et al. evaluated the expression profile of the five SSTR subtypes (SSTR1–5), including 20 meningioma tissue samples, using Northern blot analysis, reverse transcription polymerase chain reaction (RT-PCR), and immunocytochemistry [15]. Their results demonstrated that meningiomas expressed multiple SSTR subtypes with varying frequencies. RT-PCR analysis showed positivity rates of SSTR1 in 86% of tumors, SSTR2 in 100%, SSTR3 in 60%, SSTR4 in 58%, and SSTR5 in 67%. Notably, 85% of tumors co-expressed at least three of the five receptor subtypes, indicating a broad and heterogeneous receptor distribution. SSTR2 expression is also maintained in high-grade meningiomas [16]. Similarly, Silva et al. reported that through immunohistochemical expression with polyclonal antibodies all five SSTR subtypes were detectable in meningiomas at frequencies ranging from 61.6% to 100%, with SSTR2 being the most predominant [17]. Of interest, they found that, in addition to tumor-cell expression, SSTR1 and SSTR2 were also detected in endothelial cells. A large immunohistochemical study involving 726 meningioma samples systematically analyzed the expression profiles of SSTR1-5. The results demonstrated that SSTR1, SSTR2A, and SSTR5 were highly expressed overall, while significant heterogeneity in SSTR expression was observed among different clinical subgroups [18].

The diagnostic utility of SSTR-based SPECT was first assessed by Klutmann et al. studying 27 meningioma patients before and after surgery. While [^111^In]In-octreotide and MRI imaging were positive in all 27 patients, the postoperative follow-up MRI demonstrated diffuse contrast enhancement post-treatment, not allowing differentiation between scarring and oedema and recurrence. Inversely, on [^111^In]In-octreotide imaging, 16 of the 27 patients demonstrated focal increased tracer uptake in the post-treatment bed concordant with recurrence [19]. Nathoo et al., using [^111^In]In-pentetreotide, reported a sensitivity, specificity, positive predictive value, and negative predictive value of 100%, 50%, 75%, and 100%, respectively, for the detection of meningiomas [20]. The current recommended use of SSTR PET/CT imaging in meningioma is based on meningioma tumor tissue analysis as well as on imaging data. A recent systematic review including 14 PET studies, 8 using [^68^Ga]Ga-DOTATATE, 5 using [^68^Ga]Ga-DOTATOC and 1 using [^68^Ga]Ga-DOTANOC, encompassing 534 patients and 733 meningiomas, summarized the role of SSTR-targeted PET in meningiomas [21]. The review highlighted the complementary value of SSTR-targeted PET to MRI for meningioma detection, assessment of osseous involvement, and differentiation of recurrent tumor from post-treatment changes, while noting heterogeneity in study methods and reference standards. More recently, Unterrainer et al. evaluated [^18^F]F-SiTATE in 107 patients with known or suspected meningioma [22]. This showed excellent tumor-to-background contrast, with higher uptake in meningioma lesions than in non-meningioma lesions (SUVmax 11.6 ± 10.6 vs. 4.0 ± 3.3, *p* < 0.001), and enabled the detection of previously unrecognized tumor sites and osseous involvement. Notably, 59 of 231 lesions (25.6%) identified on PET/CT had not been reported on standard imaging.

The role of PRRT using ^177^Lu- or ^90^Y-labeled somatostatin analogues in patients suffering from advanced, refractory, or unresectable SSTR-positive meningioma has been recently reviewed by Gujral et al. [23]. A recent prospective multicenter phase II study (NCT03971461) by Kurz et al. evaluated [^177^Lu]Lu-DOTATATE (7.4 GBq every 8 weeks for 4 cycles) in 14 patients with progressive intracranial meningioma who had previously undergone surgery and at least one course of radiotherapy [24]. Treatment was well tolerated, with no grade 3-4 renal or hepatic toxicity. Seven patients (50%) achieved PFS at 6 months, meeting the predefined threshold and allowing continued trial enrollment; stable disease was the best radiographic response in all seven patients. Notably, a >25% reduction in [^68^Ga]Ga-DOTATATE uptake was observed in 5 meningioma lesions from 2 patients, suggesting that SSTR-PET may serve as a useful imaging biomarker for therapeutic response. The role of PRRT for the treatment of meningioma is currently being evaluated in the LUMEN-1 trial (NCT06326190). Of interest, more recently, Amerein et al. performed intra-arterial PRRT in 13 patients suffering from advanced meningioma, with 10 patients showing a radiological response or stable disease, supporting the feasibility and safety of this approach [25].

#### 2.1.2. Glioma

Gliomas are the most common primary malignant tumors of the central nervous system, characterized by diffuse infiltration, therapeutic resistance, and poor prognosis [13]. Therefore, there is an urgent need for more effective diagnostic and therapeutic strategies. Reubi et al. were the first to demonstrate that SSTRs are expressed in glial tumors using autoradiographic techniques or in vitro binding assays with membrane homogenates [26]. Subsequently, Mawrin et al. illustrated the heterogeneity of SSTR expression through immunohistochemistry using polyclonal rabbit antibodies and Western blot analysis [27]. In diffuse astrocytomas, SSTR1 and SSTR2 were absent, and only occasional expression was seen in anaplastic astrocytomas. In glioblastoma, SSTR1 was present in 66% of tumors, and SSTR2 in 44%. Expression of SSTR3 was detected in 38% of diffuse astrocytoma, 40% of anaplastic astrocytomas, and 84% of glioblastoma multiforme. Expression of SSTR4 and SSTR5 was observed in 80% and 25% of all cases, respectively, and did not vary with histological grade. Then studies using improved antibodies and modern molecular classification systems have refined these observations. Immunohistochemical analyses using the monoclonal antibody UMB-1 by Kiviniemi et al. suggest that astrocytomas and glioblastomas generally show minimal SSTR2A expression, with no prognostic significance, whereas oligodendrogliomas may display stronger membranous and cytoplasmic staining [8]. Lange et al. further evaluated SSTR expression in 57 grade I–IV astrocytic tumor samples using subtype-specific rabbit monoclonal antibodies [28]. SSTR expression in astrocytic tumors was generally low, with positivity rates of 25% for SSTR2, 46% for SSTR3, and 84% for SSTR5, while SSTR1 was absent. Of interest, SSTR expression was prominent in glioma-associated tumor capillaries. SSTR2, SSTR3, and SSTR5 were detected on tumor microvessels in 37%, 44%, and 96% of cases, respectively, suggesting that vascular expression, particularly of SSTR5, may represent an important feature of glioma biology. Recent immunohistochemical analyses comparing benign central nervous system tissues with gliomas demonstrated reduced SSTR2 protein expression in glioma specimens, together with increased immune-cell infiltration, suggesting that loss of SSTR2 may accompany malignant transformation and microenvironmental remodeling in gliomas [29].

Functional imaging studies have yielded mixed results. Early investigations using ^111^In-labeled somatostatin analogs demonstrated that some high-grade gliomas exhibit SSTR tracer accumulation. In a series by Luyken et al. including 19 patients, 8 suffering from grade II glioma and 11 suffering from grade III or IV glioma, high focal uptake was found in all grade III and IV gliomas whereas none of the grade II glioma demonstrated tracer uptake [30]. Kiviniemi et al. evaluated SSTR-targeted PET in 27 patients with primary or recurrent high-grade gliomas, using [^68^Ga]Ga-DOTANOC in 24 patients and [^68^Ga]Ga-DOTATOC in the first 3 patients [31]. Tracer uptake was observed in all 19 tumors with disrupted blood–brain barrier (BBB), whereas no uptake was detected in tumors with an intact BBB. These findings suggest that PET signal in gliomas may be influenced by increased vascular permeability and passive tracer extravasation, although the relative contributions of receptor-mediated binding and nonspecific uptake remain uncertain.

Despite these limitations, therapeutic applications have been explored. Early clinical studies demonstrated that intracavitary or intratumoral administration of [^90^Y]Y-DOTATOC achieved durable responses in selected WHO grade II–III gliomas, suggesting the potential feasibility of locoregional SSTR-targeted radiopeptide therapy [32]. In a pilot study of three patients with recurrent glioblastoma, intracavitary administration of [^90^Y]Y -DOTATOC via an implanted catheter achieved one complete and two partial radiologic responses with minimal toxicity, supporting the feasibility of locoregional SSTR-targeted radionuclide therapy [33].

### 2.2. Nasopharyngeal Carcinoma

Nasopharyngeal carcinoma (NPC) is a malignant epithelial tumor arising from the nasopharyngeal mucosa that exhibits light microscopic or ultrastructural evidence of squamous differentiation. According to the WHO Classification of Head and Neck Tumors (5th edition), NPC comprises three major histologic subtypes: non-keratinizing nasopharyngeal carcinoma (NK-NPC), keratinizing nasopharyngeal carcinoma (K-NPC), and basaloid squamous cell carcinoma [34]. Among these, NK-NPC (WHO classes II and III) is the most prevalent subtype and shows a strong etiological association with Epstein–Barr virus (EBV) infection. Although treatment outcomes have improved, patients with recurrent, metastatic, or advanced disease continue to have unfavorable survival, highlighting the need for earlier diagnosis and novel therapeutic strategies. Evidence accumulated from tissue-based studies indicates that SSTR2 is the predominant and clinically relevant SSTR subtype in NPC. Loh et al. investigated SSTR expression in NPC using receptor autoradiography with ^125^I-labeled [Tyr^3^]-octreotide and ^125^I-labeled [Leu^8^, DTrp^22^, Tyr^25^]-somatostatin-28 as radioligands [35]. Among 12 NPC biopsy specimens, 9 cases demonstrated moderate to high SSTR expression, whereas no measurable receptor expression was detected in 5 non-neoplastic nasopharyngeal tissue samples. Tao et al. further reported by immunohistochemistry using monoclonal antibody that SSTR2A was highly expressed on both primary and metastatic NK-NPC tumor cells, being positive in 93 of 99 primary NK-NPC samples (93.9%), whereas none of the K-NPC samples examined showed positive staining [9]. Additional mechanistic evidence was provided by Lechner et al., who evaluated SSTR2 expression in multiple clinical cohorts comprising 402 primary, locally recurrent, and metastatic NPC tumors [36]. Using tissue-based expression analyses together with cellular and preclinical animal models, they demonstrated that SSTR2 is highly expressed in NPC and further showed that its expression can be induced by EBV latent membrane protein 1 through activation of the NF-κB signaling pathway. In addition, [^68^Ga]Ga-DOTATATE PET/CT imaging in a clinical trial of NPC patients confirmed in vivo uptake of an SSTR2-targeting radiotracer, supporting the functional presence of SSTR2 in this malignancy.

The favorable tissue expression profile of SSTR2 has translated into encouraging molecular imaging results. Schartinger et al. studied 5 patients suffering from EBV positive NPC and found increased [^68^Ga]Ga-DOTATOC uptake in all 5 patients [37]. Khor et al. studied 4 NK-NPC patients, three presenting with a primary tumor and one patient with a recurrence using [^68^Ga]Ga-DOTANOC PET/CT imaging [38]. All three primary lesions showed a significant uptake of [^68^Ga]Ga-DOTANOC whereas the recurrence lesion showed low uptake. Zhao et al. studied 36 NK-NPC patients, 25 for initial staging and 11 for recurrence detection using [^68^Ga]Ga-DOTATATE PET/CT, the majority of which (33/36 patients) also had [^18^F]F-FDG PET/CT performed [39]. Primary lesions and involved regional lymph nodes showed a significantly higher [^68^Ga]Ga-DOTATATE uptake compared to that of [^18^F]F-FDG whereas uptake of both tracers was comparable for bone metastases and visceral lesions. [^68^Ga]Ga-DOTATATE uptake proved significantly correlated to SSTR2 expression. Moreover, preclinical studies of peptide PRRT have suggested a potential therapeutic benefit for SSTR-directed treatment strategies in NPC [40]. In C666-1 xenograft models, [^177^Lu]Lu-LNC1010 showed higher tumor uptake, prolonged retention and greater tumor growth inhibition than [^177^Lu]Lu-DOTATATE. In a pilot clinical application, PRRT with [^177^Lu]Lu-LNC1010 achieved favorable therapeutic response with negligible adverse effects in a patient with metastatic NPC.

### 2.3. Differentiated Thyroid Carcinoma

Differentiated thyroid carcinoma (DTC), primarily comprising papillary carcinoma (approximately 80%) and follicular carcinoma (10–20%), is generally associated with a favorable prognosis. However, patients with recurrent or distant metastatic disease often have a poorer outcome [41]. Notably, these advanced cases frequently exhibit reduced iodine uptake, limiting the effectiveness of radioiodine therapy and highlighting the need for alternative diagnostic and therapeutic strategies. Previous studies have reported SSTR expression in DTC. For instance, Pazaitou-Panayiotou et al. analyzed 47 patients with non-medullary thyroid carcinomas through immunohistochemistry (using polyclonal SSTR specific antibodies) and found that SSTR subtypes SSTR2 and SSTR3 were expressed in all non-medullary thyroid carcinomas, while SSTR 1 and SSTR5 were detected in 75% of cases and SSTR4 in 38% [42]. Other small-number studies, for example, Pisarek et al., have used immunohistochemistry (with rabbit polyclonal antibodies) and qRT-PCR on a mixed cohort of nine benign and malignant thyroid tumors, demonstrating SSTR1 positivity in 88.8% of cases. Expression rates for other receptor subtypes were 44.4% for both SSTR2A and SSTR2B, 55.5% for SSTR3, 11.2% for SSTR4, and 33.3% for SSTR5. Concordance between immunohistochemistry and RT-PCR was high for SSTR1, SSTR2A, and SSTR3, supporting the reliability of these findings [43]. Druckenthaner et al. assessed SSTR expression in thyroid tumors and normal thyroid tissue using RT-PCR and SSTR2 immunohistochemistry (rabbit polyclonal antibodies) [44]. Among samples from 21 patients, 94% expressed SSTR mRNA, with SSTR2 as the predominant subtype. SSTR2 immunostaining was positive in 87% of cases and correlated well with RT-PCR findings. In contrast, more recent evidence using better-validated antibodies suggests a lower prevalence of receptor expression. In a study including 90 samples from 56 patients, including papillary, follicular, medullary, and anaplastic subtypes, Czajkowski et al. demonstrated that SSTR protein expression levels were generally low via immunohistochemistry (well-characterized rabbit monoclonal antibodies) [45]. In the overall mixed cohort, SST2 was the most frequently expressed receptor, found in 38% of patients, followed by SST5 and SST4, found in 14 and 9% of patients, respectively (positivity defined as IRS ≥3). SST1 and SST3 could not be detected to any significant extent. Notably, strong expression of the SSTR was found on the tumor capillaries in many cases. Taken together, these findings indicate that SSTR expression in DTC is heterogeneous. This variability may limit its role as a universal target but supports its potential application in selected patients for molecular imaging, particularly in radioiodine-refractory disease.

Based on this biological background, SSTR-targeted scintigraphy has been explored in DTC. SSTR scintigraphy has shown particular value in detecting lesions in radioiodine-refractory disease. In 10 patients with suspected recurrence or metastasis, [^99m^Tc]Tc-depreotide imaging detected recurrent lesions in 9 patients, compared with 7 patients on FDG PET [46]. Rodrigues et al. evaluated 18 patients with DTC and elevated thyroglobulin levels, of whom 12 were radioiodine-negative. In the overall cohort, [^111^In]In-DOTA-LAN and [^111^In]In-DOTATOC detected 37 and 33 lesions, respectively, each in 17 patients, whereas [^18^F]F-FDG PET detected 30 lesions in 15 patients [47]. Collectively, these studies suggest that SSTR scintigraphy may provide complementary diagnostic information, particularly in radioiodine-refractory DTC. With the development of PET imaging, SSTR PET tracers have also been applied in DTC. Comparative studies between [^68^Ga]Ga-DOTANOC and [^18^F]F-FDG in 62 patients with elevated thyroglobulin and negative radioiodine scans have demonstrated that both modalities show comparable patient-based detection rates (65% vs. 72%), although [^18^F]F-FDG generally identifies a greater number of lesions (168 vs. 121) [48]. In patients with recurrent DTC, Middendorp et al. reported higher lesion-based detection with FDG than DOTATOC in radioiodine-positive (28/31 vs. 25/31) and radioiodine-negative subgroups (70/73 vs. 26/73; *p* < 0.01), with three lesions detected only by DOTATOC [49]. Overall, these results indicate that while SSTR PET may not surpass FDG PET in lesion-based sensitivity, it provides complementary biological information that may be valuable for patient stratification and therapeutic decision-making.

Given the presence of SSTR expression and its detectability on molecular imaging, PRRT has been investigated as a potential therapeutic option for patients with radioiodine-refractory DTC. Clinical studies have demonstrated that PRRT can achieve disease stabilization in a subset of patients, although objective response rates remain limited. For example, treatment with [^177^Lu]Lu-DOTATATE resulted in partial response in one patient, while others showed progressive disease, reflecting considerable heterogeneity in therapeutic response [50]. Similar outcomes have been reported in small patient cohorts, with cases of stable disease, minor response, and partial response observed following PRRT [51]. In addition to ^177^Lu-based therapy, ^90^Y-labeled agents have also been evaluated in advanced DTC. Treatment outcomes indicate partial response or disease stabilization in some patients, with a median overall survival of approximately 21 months following PRRT initiation [52]. Furthermore, patient selection based on SSTR imaging and FDG PET appears to be important, as functional tumor volume has been associated with treatment response [53]. Lee and Kim’s meta-analysis included 98 patients with MTC and 67 with DTC. Across six DTC studies, the pooled radiological ORR and DCR were 15.61% and 53.95%, respectively. DTC subgroup estimates for ORR/DCR were 24.52%/67.26% with [^177^Lu]Lu-based PRRT and 13.82%/50.86% with [^90^Y]Y-based PRRT. Serious adverse events occurred in 2.82% of DTC patients [54].

### 2.4. Non-Small Cell Lung Cancer

Lung cancer is one of the most common malignancies worldwide and remains the leading cause of cancer-related mortality [55]. Lung cancer is broadly classified into non-small cell lung cancer (NSCLC, 75–80%) and small cell lung cancer (SCLC, 20–25%). Despite advances in therapeutic strategies, the overall survival of patients with advanced lung cancer remains poor. Therefore, the identification of novel diagnostic and therapeutic targets remains an area of active investigation. Previous studies have reported that approximately 50% of SCLC tumors express SSTR2 [56]. The expression of SSTR in NSCLC has been investigated in several studies. Liu et al. reported that, based on immunohistochemical analysis (using polyclonal antibodies) of 62 NSCLC cases, the positivity rates of SSTR2A and SSTR5 were 48.4% (30/62) and 71.0% (44/62), respectively [57]. Stumpf et al. further characterized SSTR expression patterns in lung adenocarcinoma and squamous cell carcinoma with rabbit monoclonal antibodies [58]. SSTR5 was detected in approximately 75% of NSCLC samples. SSTR1 was present in 36.4% of ADC and 14.3% of SCC cases, whereas SSTR2 was observed in 23.8% of SCC samples. SSTR4 expression was rare, detected in approximately 4–5% of cases. In addition, strong expression of SSTR2, SSTR3, and SSTR5 was observed in tumor-associated stromal blood vessels. SSTR5 was present in the vasculature of all tumor samples, while SSTR3 was detected in more than 90% of cases, irrespective of tumor entity. These findings have led to the evaluation of SSTR-targeted radiotracers in pulmonary nodules. Consequently, both SSTR-based SPECT and PET imaging approaches have been explored in NSCLC assessment.

In the SPECT domain, Blum et al. assessed [^99m^Tc]Tc-depreotide for solitary pulmonary nodules characterization and reported a high sensitivity of 96.6% and a specificity of 73.1% among 114 patients [59]. Kahn et al. enrolled 166 patients, of whom 157 had evaluable lung lesions, to compare [^18^F]F-FDG PET with [^99m^Tc]Tc-depreotide in suspected NSCLC cases and found similar sensitivity for the primary tumor, although [^18^F]F-FDG demonstrated higher overall specificity and better staging performance [60]. Additional studies confirmed that ^99m^Tc-labeled SSTR imaging represents a viable diagnostic option in settings where PET is not available. In a mixed cohort of 44 patients, [^99m^Tc]Tc-octreotide and [^18^F]F-FDG dual-head coincidence imaging both showed 100% patient-based sensitivity (31/31) for primary tumors, whereas sensitivity for hilar/mediastinal nodal involvement was 35% (7/20) versus 100% (20/20), respectively [61]. Comparable diagnostic performance was also observed with [^99m^Tc]Tc-HYNIC-TATE, which achieved 100% sensitivity and 79% specificity in the evaluation of SPNs [62]. Further work by Wang et al. showed that [^99m^Tc]Tc-octreotide SPECT/CT could assist in differentiating benign from malignant nodules [63]. PET imaging using [^68^Ga]Ga-labeled somatostatin analogues has enabled a more refined characterization of SSTR expression in NSCLC. In a dynamic [^68^Ga]Ga-DOTATOC PET study of nine NSCLC patients, Dimitrakopoulou-Strauss et al. reported mild to moderate uptake in primary tumors, with a mean SUV of approximately 2.0, substantially lower than the mean SUV of FDG at 5.7 [64]. Although some quantitative parameters were correlated between [^68^Ga]Ga-DOTATOC and [^18^F]F-FDG, all FDG-positive metastatic lesions were DOTATOC-negative, suggesting loss of SSTR2 expression during tumor progression. Consistent findings were observed in a prospective study of thirty patients with 31 pulmonary lesions who underwent both [^68^Ga]Ga-DOTATATE and [^18^F]F-FDG PET/CT [65]. Overall diagnostic accuracy was similar for the two tracers. However, for lesion-based differentiation of benign and malignant pulmonary lesions, [^68^Ga]Ga-DOTATATE demonstrated greater specificity (94% vs. 81%) and lower sensitivity (73% vs. 93%) than FDG. Notably, [^68^Ga]Ga-DOTATATE uptake correlated with SSTR2 expression in stromal rather than epithelial components, emphasizing the role of the tumor microenvironment in determining SSTR imaging behavior.

### 2.5. Breast Cancer

Breast cancer is the second most diagnosed malignancy worldwide and remains a leading cause of cancer-related mortality among women [66]. Although endocrine therapy, cytotoxic agents, and targeted therapies are widely used, patients with advanced or metastatic estrogen receptor-positive disease often experience suboptimal clinical outcomes. Increasing attention has been directed toward SSTRs as potential theranostic targets in breast cancer. Previous studies have investigated SSTR expression in breast cancer cell lines and tumor tissues at both the mRNA and protein levels, including immunohistochemistry analyses, and have consistently shown that receptor expression is heterogeneous across studies and tumor subtypes [67,68]. In a representative analysis of 98 breast tumor samples, Kumar et al. reported variable expression of all five SSTR subtypes through RT-PCR and immunohistochemistry (polyclonal antibodies and antigen-adsorbed antibodies), with positivity rates of 91% for SSTR1, 98% for SSTR2, 96% for SSTR3, 76% for SSTR4, and 54% for SSTR5 [69]. Interestingly, SSTR2 and SSTR5 showed prominent vascular expression, whereas other subtypes displayed weaker or variable patterns. Similarly, Zou et al. reported heterogeneous expression of SSTR subtypes in 160 breast cancer tissues using immunohistochemistry (monoclonal antibodies), with positivity rates of 90.0%, 34.4%, 41.9%, 71.3%, and 44.4% for SSTR1–5, respectively [10]. Additionally, in triple-negative breast cancer, approximately 28% of tumor specimens showed positive staining for SSTR2 [70]. Collectively, these findings suggest that SSTR expression in breast cancer is not uniform but appears to be enriched in a subset of tumors, particularly in hormone receptor-positive disease, thereby providing a biological basis for the subsequent exploration of SSTR-targeted imaging approaches.

Early investigations using SSTR-based SPECT demonstrated variable detection of breast cancer lesions. These studies employed [^111^In]In-pentetreotide, [^111^In]In-octreotide [71], [^99m^Tc]Tc-depreotide [72], and [^99m^Tc]Tc-octreotide and yielded heterogeneous results, reflecting differences in receptor density and imaging sensitivity. Initial clinical evidence demonstrated that SSTR-based scintigraphy was capable of visualizing primary breast tumors. For example, van Eijck et al. reported that 39 of 52 primary breast cancers were detected using [^111^In]In-pentetreotide, corresponding to a detection rate of 75% [11]. Albérini et al. evaluated [^111^In]In-pentetreotide in 13 patients with suspected breast cancer [73]. Among 11 patients with confirmed malignant primary tumors, 4 of 11 primary tumors were visualized. However, among 6 metastatic axillary lymph nodes evaluated, only 2 were detected, suggesting limited performance for the detection of nodal metastases. Van den Bossche et al. reported that, in 20 patients with advanced breast cancer, baseline [^99m^Tc]Tc-depreotide scintigraphy combined with change in tracer uptake after one cycle of endocrine therapy predicted clinical response with an accuracy of 100% among the 18 evaluable patients [72]. These results suggested a potential role for SSTR scintigraphy in functional assessment. Wang et al. conducted a prospective study in 55 patients and reported that the sensitivity, specificity, positive predictive value, and negative predictive value of [^99m^Tc]Tc-octreotide for primary lesion detection were 91.8%, 22.2%, 70.8%, and 57.1%, respectively [74]. However, the authors noted that non-specific uptake in normal breast tissue and low tumor-to-background contrast limited the accuracy of detecting axillary lymph node metastases. A significant correlation was observed between tracer uptake and SSTR2 mRNA expression. The limitations of SPECT-based SSTR imaging in breast cancer have prompted subsequent investigations using PET imaging, which provides higher spatial resolution and enables quantitative assessment. Recent clinical studies have examined the performance of SSTR-targeted PET in estrogen receptor-positive and progesterone receptor-positive breast cancers, which may show relatively low glycolytic activity on [^18^F]F-FDG imaging. In the first head-to-head comparison of [^68^Ga]Ga-DOTATATE and [^18^F]F-FDG PET/CT in metastatic estrogen receptor positive/ progesterone receptor-positive/Human epidermal growth factor receptor 2-negative disease, Nguyen et al. reported that eight of ten patients had identifiable lesions with both tracers [75]. Considerable interlesional heterogeneity was observed. Several bone metastases were [^68^Ga]Ga-DOTATATE-positive but [^18^F]F-FDG-negative, whereas most visceral lesions were [^18^F]F-FDG-positive with minimal [^68^Ga]Ga-DOTATATE uptake. These findings indicate heterogeneity of SSTR expression among metastatic sites, although further studies are needed to confirm these observations. In a larger cohort study, Chandekar et al. evaluated paired [^18^F]F-FDG and [^68^Ga]Ga-DOTATATE PET/CT in ER-positive metastatic breast cancer and found that the overall per-patient detection rates were comparable [76]. However, [^68^Ga]Ga-DOTATATE detected fewer visceral/other metastatic lesions than [^18^F]F-FDG (128 vs. 235; *p* = 0.003) and yielded a lower PET-based M-stage in 10% of patients. Only a small proportion of cases, approximately 10%, reached Krenning scores of 3 or higher, suggesting that eligibility for SSTR-targeted radionuclide therapy may be limited to a small subgroup of patients. In a prospective phase II clinical trial (NCT05880394), SSTR2-targeted DOTATATE PET/CT was evaluated in patients with biopsy-proven metastatic estrogen receptor-positive breast cancer [77]. A total of 30 female participants underwent [^68^Ga]Ga-DOTATATE PET/CT imaging. A positive scan was defined by the presence of lesions with a Krenning score of 3 or 4. Among the enrolled patients, 9 of 30 (30%) showed high tracer uptake corresponding to Krenning scores of 3–4, indicating elevated SSTR2 expression in a subset of metastatic lesions. Among these, a single patient with Krenning score 4 lesions received [^225^Ac]Ac-DOTATATE as a proof-of-concept therapeutic intervention. This represents an isolated case and further prospective evaluation in larger cohorts is needed before any conclusions on therapeutic efficacy can be drawn.

### 2.6. Lymphoma

Lymphoma is one of the most common hematologic malignancies worldwide and given the persistent unmet needs in relapsed or aggressive disease, SSTR expression may provide a rationale for novel molecular imaging and targeted radionuclide therapy strategies [78]. However, overall levels are substantially low and vary considerably among histologic subtypes. Dalm et al. demonstrated using RT-PCR and quantitative PCR that SSTR2 and SSTR3 mRNA levels are low in lymphomatous tissues, including orbital lymphomas, Hodgkin lymphoma, and non-Hodgkin lymphoma [79]. These findings are consistent with autoradiographic analyses showing weak binding of radiolabeled octreotide, as well as negative immunohistochemical staining (using polyclonal antibody with peptide-blocking controls) for SSTR2 and SSTR3. In addition, lymphoid cell lines exhibit only a limited number of high-affinity somatostatin binding sites, with correspondingly low in vitro uptake. Despite these overall low expression levels, larger immunohistochemical studies indicate that SSTR expression is not entirely absent but follows a subtype-specific pattern. In a cohort of 103 lymphomas, Juntikka et al. reported SSTR2 immunopositivity (rabbit monoclonal antibodies) in approximately 46 to 56% of diffuse large B-cell lymphoma, Hodgkin lymphoma, and follicular lymphoma, with expression most frequently localized to the cell membrane [80]. It is worth noting that strong SSTR immunoreactivity in some lymphoma specimens was predominantly localized to non-neoplastic background cells rather than tumor cells. SSTR2 expression was consistently observed in macrophages, neutrophils, follicular dendritic cells, and endothelial cells, whereas SSTR3 was prominent in vascular endothelial cells, macrophages, mast cells, and fibroblasts. SSTR3 and SSTR5 were rarely observed in diffuse large B-cell lymphoma and follicular lymphoma but were detected in a subset of Hodgkin lymphoma cases, whereas mucosa-associated lymphoid tissue (MALT) lymphoma demonstrated modest expression across multiple SSTR subtypes. Consistently, Stollberg et al. reported that through immunohistochemistry (rabbit monoclonal antibodies) SSTR5 was the most frequently expressed subtype (approximately 50%), followed by SSTR3, SSTR2A, SSTR4, and SSTR1, with expression rates of 35%, 27%, 18%, and 2%, respectively [81]. Notably, gastric lymphomas exhibited significantly higher expression of SSTR3, SSTR4, and SSTR5 compared with extragastric tumors. Collectively, these findings indicate that SSTR expression in lymphoma is characterized by overall low levels but marked subtype-dependent heterogeneity.

Despite the marked heterogeneity of SSTR expression across different lymphoma subtypes, several studies have explored the feasibility of SSTR-based imaging in this disease. Early investigations using [^111^In]In-pentetreotide in Hodgkin lymphoma and non-Hodgkin lymphoma demonstrated that lymphomatous lesions can be detected safely; however, tracer uptake was highly variable and generally lower than that observed in neuroendocrine tumors [82]. In a prospective study including 35 patients with Hodgkin and non-Hodgkin lymphoma, [^111^In]In-pentetreotide was used for staging, but concordance with clinical staging was limited, with only 10 of 35 patients correctly staged, while 22 were understaged and 3 overstaged scintigraphically [83]. In a larger cohort comprising 47 patients with Hodgkin lymphoma and 59 with various subtypes of non-Hodgkin lymphoma, [^99m^Tc]Tc-depreotide demonstrated higher lesion detection rates compared with ^67^Ga scintigraphy and showed relatively higher uptake in Hodgkin lymphoma and indolent lymphomas [84]. During initial staging, [^99m^Tc]Tc-depreotide identified most supra-diaphragmatic and inguinal lymph nodes, as well as all cases with splenic involvement; however, sensitivity for abdominal lymph nodes (22.7%), liver (45.5%), and bone marrow involvement (36.4%) remained limited. Post-therapy imaging correctly identified most cases of refractory or recurrent disease, although specificity was moderate due to inflammatory or nonspecific uptake. In contrast, in extranodal mucosa-associated lymphoid tissue lymphoma, Raderer et al. reported consistently positive [^111^In]In-pentetreotide uptake at sites of confirmed disease, supporting its applicability for staging and treatment monitoring in this subtype [85]. A comparative study evaluating [^67^Ga]Ga scintigraphy, [^111^In]In-DOTA-TOCT, and [^111^In]In-DOTA-LAN further demonstrated the feasibility of SSTR-targeted imaging in MALT lymphoma [86]. ^67^Ga scintigraphy detected abnormal uptake in 63% of patients and 58% of involved sites, while [^111^In]In-DOTA-TOCT and [^111^In]In-DOTA-LAN showed comparable detection rates, detecting 56% (15/27) and 55% (12/22) of lesions, with occasional false-positive findings observed across all tracers. Collectively, these studies demonstrate that SSTR-targeted imaging in lymphoma is feasible but characterized by variable detection performance across tracers and disease subtypes. Given the generally low and heterogeneous expression of SSTR in lymphoma, evidence regarding SSTR-targeted radionuclide therapy in this disease remains limited.

### 2.7. Melanoma

Melanoma is one of the most aggressive forms of skin cancer, with a rapidly increasing incidence worldwide [87]. Despite advances in systemic therapies, metastatic melanoma remains associated with substantial morbidity and mortality. Accurate detection of metastatic disease and identification of suitable therapeutic targets remain critical for disease management. SSTRs have been reported in melanoma. Lum et al. analyzed twenty-three melanoma samples from seventeen patients using RT-PCR with subtype-specific primers and confirmed the amplified products by Southern blot [12]. Their findings demonstrated high rates of SSTR expression across tumors, with positivity for SSTR1 in 96%, SSTR2 in 83%, SSTR3 in 61%, SSTR4 in 57%, and SSTR5 in 9% of melanoma samples. Among sixteen patients who underwent preoperative OctreoScan, 63% of tumors were visualized, supporting the presence of SSTRs in melanoma and indicating that SSTR scintigraphy can detect a subset of lesions.

Further clinical evaluation by Valencak et al. using [^111^In]In-DOTA-lanreotide and [^111^In]In-DOTA-Tyr^3^-octreotide in 23 patients with stage IV melanoma showed visualization of 62% (38/61) and 43% (10/23) of metastatic lesions, respectively [88]. However, in vitro studies demonstrated no functional SSTR expression or antiproliferative response to somatostatin analogues in melanoma cell lines. These findings raise the possibility that the in vivo tracer signal may partly arise from non-melanoma cellular components within the tumor microenvironment, including vascular or immune-associated elements. However, this interpretation remains hypothetical, as the available studies did not directly localize SSTR expression to specific stromal, vascular, or immune-cell populations in melanoma tissue.

A PET/CT study including 18 patients employing [^68^Ga]Ga-DOTATOC further highlighted the limitations of SSTR-targeted imaging in melanoma [89]. Although 61% of patients demonstrated [^68^Ga]Ga-DOTATOC-positive metastases, only 22% of [^18^F]F-FDG–avid lesions were confirmed on a per-lesion basis. [^68^Ga]Ga-DOTATOC uptake was generally faint, with a mean SUVmax of 3.1 compared with 28.2 for FDG. These results indicate that [^68^Ga]Ga-DOTATOC is unlikely to provide effective lesion detection or therapeutic targeting for metastatic melanoma.

## 3. Discussion

SSTR expression across non-NETs exhibits a highly heterogeneous landscape that differs from the generally higher and more homogeneous receptor expression observed in gastroenteropancreatic neuroendocrine tumors [90]. As synthesized in this review, SSTR expression in non-NETs is neither rare nor uniform but instead reflects a spectrum of biological contexts. Importantly, SSTR signals may originate not only from tumor cells but also from tumor-associated vasculature, immune infiltrates, and stromal components. Consequently, the clinical interpretation of SSTR-targeted imaging in non-NETs requires integration of tumor type, cellular source of expression, and specific clinical context, rather than a direct extrapolation from NET paradigms.

Based on the available evidence, SSTR expression and tracer uptake in non-NETs may be interpreted within three broad biological patterns: tumor cell–dominant expression, microenvironment-associated expression, and uptake influenced by non-receptor-specific mechanisms. Tumors such as meningioma, NK-NPC, and a subset of estrogen receptor–positive breast cancers demonstrate relatively strong tumor-cell SSTR2 expression and are therefore closer to classical receptor-driven imaging models [9,17,77]. In contrast, tumors including NSCLC and DTC more frequently exhibit SSTR signals derived from TAMs, CAFs, or vascular endothelium [45,65]. Gliomas represent a distinct category in which blood–brain barrier disruption may substantially contribute to tracer accumulation, although the relative contributions of receptor-mediated binding versus non-specific uptake remain incompletely defined. The available tissue-based studies of SSTR expression and cellular localization across non-NETs are summarized in Table 1.

The marked heterogeneity in imaging findings across studies can be attributed to several factors. First, differences in SSTR subtype distribution are critical, as most clinically used tracers preferentially target SSTR2, while other subtypes (e.g., SSTR1 or SSTR5) may predominate in certain tumors. Second, the cellular localization of receptor expression—particularly membranous versus cytoplasmic staining—affects tracer accessibility and binding. Third, intertumoral and intratumoral heterogeneity, including differences between primary and metastatic lesions, further complicates interpretation. In addition, variability in radiotracer characteristics (e.g., affinity profiles of DOTATATE, DOTATOC, and SPECT-based agents) contributes to inconsistent imaging results across studies. The different receptor affinity profiles of selected SSTR-targeting ligands are summarized in Table 2 [91,92,93]. While [^68^Ga]Ga-DOTATATE exhibits the highest affinity for SSTR2 (IC_50_ = 0.2 nM), [^68^Ga]Ga-DOTANOC shows a broader affinity spectrum, including SSTR3 (40 nM) and SSTR5 (7.2 nM). Theoretically, based on its broader in vitro affinity profile, [^68^Ga]Ga-DOTANOC may offer advantages over [^68^Ga]Ga-DOTATATE for imaging tumors that predominantly express SSTR3 or SSTR5. However, this remains a theoretical consideration derived from in vitro data and requires direct clinical comparative validation. Importantly, SSTR3 or SSTR5 expression should not be interpreted as equivalent to high membranous SSTR2 expression when an SSTR2-preferring radiopharmaceutical is used. Importantly, immunohistochemical positivity does not necessarily translate into detectable imaging signal or therapeutically actionable receptor density, which may explain discrepancies between pathology and in vivo imaging findings.

From a clinical perspective, the relevance of SSTR-targeted imaging in non-NETs is best understood through a stratified framework. Meningioma represents the most established indication, supported by consistent high-level SSTR2 expression and robust clinical evidence demonstrating added value of SSTR-PET in delineating disease extent, detecting recurrence, and guiding radiotherapy planning [94]. In contrast, tumors such as radioiodine-refractory differentiated thyroid carcinoma, NK-NPC, and a subset of ER-positive breast cancer show complementary value, particularly in clinical scenarios where conventional imaging is limited [39,48,49,75]. Clinical studies of SSTR-targeted imaging across non-NET tumor types are summarized in Table 3. In these settings, SSTR imaging should be regarded as an adjunct rather than a replacement for established modalities such as FDG PET. Conversely, in tumors including glioma, lymphoma and melanoma current evidence remains insufficient to support routine clinical application, largely due to low receptor density, non-specific uptake, or inconsistent imaging performance.

These biological distinctions also define the realistic boundaries of SSTR-based theranostics beyond NETs. The available PRRT evidence in non-NETs is summarized in Table 4. PRRT has demonstrated the most consistent benefit in meningioma, where patient selection using SSTR-PET is well established and disease control rates of approximately 50–70% have been reported [94]. In thyroid malignancies, particularly radioiodine-refractory disease, PRRT may provide disease stabilization in selected patients, although response rates remain modest and heterogeneous [54]. Similarly, in ER-positive metastatic breast cancer, SSTR-PET can identify a small subset of patients with sufficiently high receptor expression for potential therapeutic targeting [76]. However, outside these contexts, the predictive relationship between SSTR imaging positivity and PRRT efficacy remains largely undefined. Although it has been hypothesized that PRRT could exert effects through targeting SSTR-expressing stromal or immune components, this concept remains preliminary and requires further validation.

In most non-NETs, SSTR imaging should be interpreted as complementary to, rather than competitive with, established metabolic imaging modalities such as FDG PET. While FDG reflects tumor glycolytic activity and overall aggressiveness, SSTR imaging provides insight into receptor expression, differentiation status, and, in many cases, microenvironmental biology. The combination of these modalities may therefore offer a more comprehensive characterization of tumor phenotype than either approach alone.

Despite growing interest, several important limitations must be acknowledged. Current evidence is largely derived from retrospective, single-center studies with small sample sizes and substantial heterogeneity in study design. Differences in radiotracer selection, imaging protocols, and interpretation criteria hinder cross-study comparability and preclude standardized thresholds for clinical decision-making. In addition, pathologic–imaging correlation is often incomplete, particularly in tumors where SSTR expression is predominantly stromal or immune-derived. Notably, the lack of standardized assessment of receptor localization (e.g., membranous versus stromal expression) further complicates interpretation and patient selection. Furthermore, outside meningioma and selected thyroid cancer cohorts, robust predictors of response to PRRT remain undefined, and optimal treatment protocols have yet to be established.

Future research should focus on clarifying the cellular sources and biological relevance of SSTR expression through integration of molecular imaging, quantitative pathology, and genomic profiling. Multi-tracer imaging strategies combining SSTR imaging with FDG or immune-targeted tracers (e.g., PD-L1 or CD8) may provide a more comprehensive assessment of tumor immunobiology. In parallel, the development of novel radiopharmaceuticals may offer alternative strategies for tumors with low receptor density, although significant challenges remain. SSTR antagonists can recognize a greater number of receptor binding sites than agonists and have demonstrated higher tumor uptake and prolonged tumor retention in preclinical studies despite limited receptor internalization [95,96]. However, adequate receptor-mediated uptake remains a prerequisite for delivering cytotoxic radiation doses, and low receptor density therefore continues to limit the applicability of both agonist- and antagonist-based approaches. For example, [^68^Ga]Ga-SSO-120 (satoreotide trizoxetan), a novel SSTR2-targeting antagonist that binds to both active and inactive receptor conformations, has shown promising results in small cell lung cancer, with tumor detection in all patients and tumor-to-liver ratios >1 in 90.3% of cases [97]. Although SCLC is classified as a neuroendocrine carcinoma, this example is included to illustrate the potential of antagonist-based tracers in a somatostatin receptor-expressing thoracic malignancy, which may inform analogous investigations in non-neuroendocrine settings. Alpha-emitting agents, such as ^225^Ac-labeled somatostatin analogues, offer potential advantages because of their high linear energy transfer and short tissue penetration range and may therefore be particularly relevant for micrometastatic or small-volume disease with sufficient target uptake [98]. However, renal and hematologic toxicities associated with α-therapy remain important safety considerations, and clinical experience remains limited largely to neuroendocrine tumors, with no established protocols for non-NETs. Both strategies are currently investigational and require careful patient selection, prospective validation, and optimized dosimetry before clinical implementation in non-neuroendocrine tumors. For tumors with predominantly microenvironment-driven uptake, rational combination strategies integrating PRRT with immunotherapy or radiotherapy warrant investigation.

## 4. Conclusions

SSTR expression in non-neuroendocrine tumors is biologically diverse and context-dependent. Rather than representing a uniform receptor-driven phenomenon, SSTR imaging in non-NETs reflects a continuum ranging from tumor-cell–dominant expression to microenvironment-driven uptake. This distinction is critical for accurate interpretation of imaging findings and for defining the appropriate clinical role of SSTR-targeted strategies. With continued refinement of patient selection, imaging techniques, and therapeutic approaches, SSTR-based imaging and theranostics may evolve into a valuable, microenvironment-aware platform across selected tumor types.

## Figures and Tables

**Table 1:** SSTR expression and cellular localization across non-neuroendocrine tumors.

Tumor Type	Study	Samples (*n*)	SSTR Subtype(s)	Detection Method/Antibody	Positivity (%)	Expression Site	Main Findings
Meningioma	Dutour et al. [15]	20 meningiomas and 9 gliomas	SSTR1–5	IHC and RT-PCR	Mixed cohort: SSTR1: 86%, SSTR2: 100%, SSTR3: 60%, SSTR4: 58%, SSTR5: 67%	Tumor cells	SSTR2 is the most abundant isoform; 85% of meningiomas co-express ≥3 subtypes.
	Barresi et al. [16]	35	SSTR2A	IHC	74.3%	Tumor cells	SSTR2A expression correlates with neo-angiogenesis, supporting SSTR-targeted therapy in meningiomas.
	Silva et al. [17]	60	SSTR1–5	IHC with polyclonal antibodies	SSTR1: 61.6%; SSTR2: 100%; SSTR3: 80.6%; SSTR4: 68.3% SSTR5: 78.3%	Tumor cells; SSTR1 and SSTR2 also detected in endothelial cells	SSTR5 shows higher expression in benign tumors; endothelial SSTR1/2 suggests vascular uptake mechanisms.
	Behling et al. [18]	726	SSTR1–5	Tissue microarray + IHC	Overall high expression of SSTR1, SSTR2A, and SSTR5	Tumor cells	SSTR1, SSTR2A, and SSTR5 showed relatively high expression, with heterogeneous expression patterns across clinicopathologic subgroups.
Glioma	Mawrin et al. [27]	Including 32 glioblastomas	SSTR1–5	IHC with polyclonal antibodies; Western blot	SSTR1: 66%; SSTR2A: 44%; SSTR3: 84%; SSTR4: 80%; SSTR5: 25%	Tumor cells	SSTR1/2A/3 increase with dedifferentiation; SSTR4/5 independent of grade.
	Kiviniemi et al. [8]	184 (101 GBM, 60 astrocytomas, 23 oligodendro-gliomas)	SSTR2A	IHC, monoclonal antibody UMB-1	79% (Oligo)/27% (IDH-mutant Astro)/23% (IDH-wildtype Astro)/13% (GBM)	Tumor cells	SSTR2A is enriched in oligodendrogliomas (79%) vs. astrocytomas (23–27%) and GBM (13%), suggesting histology-specific expression.
	Lange et al. [28]	57	SSTR1/2/3/5	IHC with monoclonal antibodies	SSTR2: 25%; SSTR3: 46%; SSTR5: 84%	Tumor cells and tumor microvessels	SSTR expression was prominent in tumor microvessels, particularly SSTR5, suggesting a potential vascular component of SSTR-targeted uptake.
Nasopharyngeal carcinoma	Tao et al. [9]	106 primary and 41 metastases	SSTR2	IHC with monoclonal antibody	Primary NK-NPC: 93.9% (93/99); primary K-NPC: 0% (0/7). Metastatic NK-NPC showed concordant expression with corresponding primary lesions.	Tumor cells	SSTR2A was expressed in 93/99 (93.9%) NK-NPC cases and was detected in both primary and metastatic tumor cells.
	Lechner et al. [36]	402 primary, locally recurrent, and metastatic NPC tumors	SSTR2	IHC primarily with rabbit monoclonal antibody UMB1	Primary NPC: 81% (252/311)	Tumor cells	SSTR2 is highly expressed in EBV-associated nasopharyngeal carcinoma and shows significant potential for molecular imaging, targeted therapy, and prognostic evaluation.
Thyroid tumors	Pazaitou-Panayiotou et al. [42]	47	SSTR1–5	IHC using polyclonal antibodies	SSTR1: 75%; SSTR2: 100%; SSTR3: 100%; SSTR4: 38%; SSTR5: 75%;	Tumor cells	Non-medullary thyroid carcinomas frequently express multiple SSTR subtypes, supporting PRRT in RAI-refractory disease.
	Pisarek et al. [43]	9	SSTR1–5	IHC with polyclonal antibodies and RT-PCR	SSTR1: 88.8%; SSTR2A: 44.4%; SSTR3: 55.5%; SSTR4: 11.2%; SSTR5: 33.3%	Tumor cells	somatostatin multiligand analogs or selective SSTR 1 agonists may be used in thyroid tumors treatment.
	Druckenthaner et al. [44]	21	SSTR2	RT-PCR and SSTR2 IHC with polyclonal antibodies	94% expressed SSTR mRNA; SSTR2 IHC positive in 87%	Tumor cells	SSTR2 is highly expressed in thyroid tumors, supporting the use of octreotide-based imaging and targeted therapy.
	Czajkowski et al. [45]	90 samples from 56 patients (mixed PTC/FTC/MTC/ATC)	SST1–5	IHC with monoclonal antibodies	Overall mixed cohort, patient-level analysis (IRS ≥ 3): SST2: 38%; SST4: 9%; SST5: 14%	Tumor cells and tumor capillaries	overall SSTR expression low in mixed cohort; vascular SSTR may contribute to imaging signals.
NSCLC	Liu et al. [57]	62	SSTR2A, SSTR5	IHC using polyclonal antibodies	SSTR2A: 48.4%; SSTR5: 71.0%	Tumor cells	SSTR2A and SSTR5 are upregulated in NSCLC and may serve as potential biomarkers for metastasis, tumor staging, and prognosis.
	Stumpf et al. [58]	22 ADC and 21 SQC	SSTR1–5	IHC with rabbit monoclonal antibodies	SSTR1: 36.4% (ADC) and 14.3% (SQC); SSTR2: 23.8% (SQC); SSTR4: 4–5%; SSTR5: 75%	Tumor cells and stromal blood vessels	Low tumor-cell SSTR expression; strong vascular SSTR2/3/5 expression suggests stromal-dominant SSTR biology.
BC	Evans et al. [67]	51	SSTR1–5	mRNA expression analysis	SSTR2 was widely expressed	Whole-tumor specimen	SSTR2 is widely expressed in breast cancer, supporting the potential therapeutic application of SSTR2-targeted somatostatin analogues.
	Schulz et al. [68]	33	SST1, SST2A, SST2B, SST3	IHC with subtype-specific antibodies	SSTR1: 52%; SSTR2: 85%; SST3: 48%	Tumor cells	Breast carcinomas frequently express SSTR2, with lower expression of SSTR1 and SSTR3, supporting SSTR-targeted therapy.
	Kumar et al. [69]	98	SSTR1–5	RT-PCR and IHC using polyclonal and antigen-adsorbed antibodies	SSTR1: 91%; SSTR2: 98%; SSTR3: 96%; SSTR4: 76%; SSTR5: 54%	Tumor cells and blood vessels	Differential expression of SSTR subtypes in breast tumors is associated with histological and hormone receptor status, suggesting potential applications in receptor-targeted diagnosis and therapy.
	Zou et al. [10]	160	SSTR1–5	IHC with monoclonal and polyclonal antibodies	SSTR1: 90.0%; SSTR2: 34.4%; SSTR3: 41.9%; SSTR4: 71.3%; SSTR5: 44.4%	Tumor cells	SSTR1 and SSTR4 are the predominant SSTR subtypes in breast cancer and may mediate tumor cell cycle arrest through receptor dimerization.
Lymphoma	Dalm et al. [79]	10 (6 orbital lymphomas, 2 Hodgkin lymphomas, 2 non-Hodgkin lymphomas)	SSTR2, SSTR3	RT-PCR, IHC with polyclonal antibody and peptide-blocking controls	Low mRNA levels; negative IHC for SSTR2/3	Whole-tumor specimens	Lymphomas show low SSTR density, their radiosensitivity suggests potential feasibility for SSTR-targeted radionuclide therapy.
	Juntikka et al. [80]	103	SSTR2, SSTR3, SSTR5	IHC with monoclonal antibodies	SSTR2 immunopositivity approximately 46–56% in DLBCL, HL, and FL	Tumor cells	SSTR2 is variably expressed in lymphomas, with some patients showing high receptor expression that may support future receptor-targeted therapies.
	Stollberg et al. [81]	55 MALT type lymphoma	SSTR1–5	IHC with monoclonal antibodies	SSTR5: 50%; SSTR3: 35%; SSTR2A: 27%; SSTR4: 18%; SSTR1: 2%	Tumor cells	SSTR expression levels in MALT lymphoma are low, suggesting limited utility for SSTR-targeted approaches in this subtype.
Melanoma	Lum et al. [12]	23 melanoma samples from 17 patients	SSTR1–5	RT-PCR with subtype-specific primers; Southern blot confirmation	SSTR1: 96%; SSTR2: 83%; SSTR3: 61%; SSTR4: 57%; SSTR5: 9%	Whole-tumor specimen	High rates of SSTR mRNA expression were detected, supporting the presence of SSTR transcripts in melanoma.
	Valencak et al. [88]	Melanoma cell lines/stage IV melanoma context	Functional SSTR expression	In vitro functional assays	No functional SSTR expression	Tumor cell lines	In vitro findings did not confirm functional SSTR expression or antiproliferative response to somatostatin analogues.

Note: IHC, immunohistochemistry; RT-PCR, reverse transcription polymerase chain reaction; GBM, glioblastoma multiforme; ADC, adenocarcinoma; SQC, squamous cell carcinoma; DLBCL, diffuse large B-cell lymphoma; HL, Hodgkin lymphoma; FL, follicular lymphoma; MALT, mucosa-associated lymphoid tissue; DTC, differentiated thyroid carcinoma; NPC, nasopharyngeal carcinoma; NSCLC, non-small cell lung cancer; BC, breast cancer; SSTR, somatostatin receptor; PRRT, peptide receptor radionuclide therapy; RAI, radioiodine; EBV, Epstein–Barr virus; NF-κB, nuclear factor kappa-light-chain-enhancer of activated B cells; TNBC, triple-negative breast cancer; NK-NPC, non-keratinizing nasopharyngeal carcinoma; K-NPC, keratinizing nasopharyngeal carcinoma; PTC, papillary thyroid carcinoma; FTC, follicular thyroid carcinoma; MTC, medullary thyroid carcinoma; ATC, anaplastic thyroid carcinoma; CAFs, cancer-associated fibroblasts; TAMs, tumor-associated macrophages; CNS, central nervous system.

**Table 2:** In vitro receptor affinity profiles of selected SSTR-targeting ligands.

SSTR-Targeting Ligand	SSTR1 IC_50_ (nM)	SSTR2 IC_50_ (nM)	SSTR3 IC_50_ (nM)	SSTR4 IC_50_ (nM)	SSTR5 IC_50_ (nM)	Experimental Model	Reference
[^111^In]In-DTPA-[Tyr^3^]-octreotate	>10,000	1.3 ± 0.2	>10,000	433 ± 16	>1000	Receptor autoradiography, SSTR1–5-expressing cells	Reubi et al., 2000 [91]
[^111^In]In-DTPA-octreotide (pentetreotide)	>10,000	22 ± 3.6	182 ± 13	>1000	237 ± 52	Receptor autoradiography, SSTR1–5-expressing cells	Reubi et al., 2000 [91]
[^68^Ga]Ga-DOTATATE	>10,000	0.2 ± 0.04	>1000	300 ± 140	377 ± 18	Receptor autoradiography, SSTR1–5-expressing cells	Reubi et al., 2000 [91]
[^68^Ga]Ga-DOTANOC	>10,000	1.9 ± 0.4	40 ± 5.8	260 ± 74	7.2 ± 1.6	Receptor autoradiography, SSTR1–5-expressing cells	Antunes et al., 2007 [92]
[^68^Ga]Ga-DOTATOC	>10,000	2.5 ± 0.5	613 ± 140	>1000	73 ± 21	Receptor autoradiography, SSTR1–5-expressing cells	Reubi et al., 2000 [91]
SiTATE	>10,000	1.0 ± 0.2	712 ± 507	364 ± 291	>1000	Competitive binding assay, SSTR1–5-expressing cell membranes	Wängler et al., 2022 [93]

Note: Values are IC_50_ (nM) and are reported as mean ± SEM where available. The experimental model and original reference are provided for each ligand. Because receptor affinity is influenced by peptide structure, chelator, coordinated metal, assay conditions, and experimental system, IC_50_ values obtained from different studies should not be interpreted as directly comparable.

**Table 3:** SSTR-targeted imaging across non-neuroendocrine tumor types.

	Study	Imaging Modality	Radiotracer	Patients (*n*)	Main Findings	Clinical Implication
Meningioma	Klutmann et al. [19]	SPECT	[^111^In]In-octreotide	27 patients	Both MRI and ^111^In-octreotide imaging were positive before surgery; in postoperative follow-up, ^111^In-octreotide identified focal uptake consistent with recurrence in 16 patients while MRI showed nonspecific diffuse enhancement.	^111^In-octreotide has a significant impact in postsurgical follow-up in patients with meningioma.
	Nathoo et al. [20]	SPECT	[^111^In]In-pentetreotide	50 patients	Sensitivity 100%, specificity 50%, PPV 75%, NPV 100% for detecting meningiomas.	Supports the diagnostic value of SSTR scintigraphy in cranial dural-based meningiomas.
	Filippi et al. [21]	PET/CT PET/MRI	[^68^Ga]Ga-DOTATATE, [^68^Ga]Ga-DOTATOC, [^68^Ga]Ga-DOTANOC	534 patients (733 meningiomas)	SSTR PET, particularly PET/MRI, aided detection of skull-base and parafalcine meningiomas and informed radiotherapy planning. Diagnostic methods varied, with limited histopathological validation.	Established role for SSTR PET/CT in diagnosis, surveillance, radiotherapy planning, and theranostic selection.
	Unterrainer et al. [22]	PET/CT	[^18^F]F-SiTATE	107 patients (231 lesions)	Higher uptake in meningioma than non-meningioma lesions; SUVmax 11.6 ± 10.6 vs. 4.0 ± 3.3; 59/231 lesions were not reported on standard imaging.	Next-generation SSTR PET tracer may improve detection of occult tumor sites and osseous involvement.
Glioma	Luyken et al. [30]	SPECT	[^111^In]In-DTPA-octreotide	19 patients	High focal uptake was found in all grade III/IV gliomas; no grade II glioma showed tracer uptake.	High-grade gliomas may show SSTR tracer accumulation.
	Kiviniemi et al. [31]	PET/CT	[^68^Ga]Ga-DOTANOC (*n* = 24) and [^68^Ga]Ga-DOTATOC (*n* = 3)	27 patients	Tracer uptake was observed in all 19 tumors with disrupted BBB, whereas no uptake was detected in tumors with an intact BBB. Uptake correlated poorly with tumor-cell SSTR2 expression.	PET uptake may be influenced by BBB disruption and increased vascular permeability; the relative contributions of receptor-mediated binding and passive tracer extravasation remain uncertain.
Naso-pharyngeal carcinoma	Schartinger et al. [37]	PET/CT	[^68^Ga]Ga-DOTATOC	5 patients	Increased uptake was observed in all 5 patients with EBV-positive NPC.	Supports feasibility of SSTR PET imaging in EBV-positive NPC.
	Khor et al. [38]	PET/CT	[^68^Ga]Ga-DOTANOC	4 patients	All three primary lesions showed significant uptake; the recurrent lesion did not.	SSTR PET may detect primary NK-NPC, but uptake can be variable in recurrence.
	Zhao et al. [39]	PET/CT	[^68^Ga]Ga-DOTATATE	36 patients	Primary lesions and regional lymph nodes showed significantly higher uptake than FDG; bone and visceral metastases showed comparable uptake.	^68^Ga-DOTATATE uptake correlated with SSTR2 expression, supporting SSTR2-targeted imaging in NK-NPC.
Thyroid carcinoma	Rodrigues et al. [46]	SPECT	[^99m^Tc]Tc-depreotide	10 patients	True-positive results were obtained in nine patients (90%) with ^99m^Tc-depreotide scintigraphy and in seven patients (70%) with [^18^F]F-FDG-PET.	^99m^Tc-depreotide scintigraphy shows potential value in diagnosing thyroglobulin-positive, radioiodine-negative thyroid cancer and aids individualized therapy planning.
	Rodrigues et al. [47]	SPECT	[^111^In]In-DOTA-LAN and [^111^In]In-DOTATOC	18 patients (12 radioiodine-negative)	[^111^In]In-DOTA-LAN and [^111^In]In-DOTATOC detected 37 and 33 lesions, respectively, each in 17 patients, whereas [^18^F]F-FDG PET detected 30 lesions in 15 patients.	SSTR-targeted imaging may provide complementary diagnostic information in DTC, including patients with elevated thyroglobulin and negative radioiodine imaging.
	Kundu et al. [48]	PET/CT	[^68^Ga]Ga-DOTANOC	62 patients	[^68^Ga]Ga-DOTANOC demonstrated disease in 40/62 (65%) patients, Per-patient sensitivity and specificity was 78.4% and 100%.	SSTR PET is less sensitive than ^18^F-FDG for lesion detection in radioiodine-negative DTC but aids PRRT selection.
	Middendorp et al. [49]	PET	[^68^Ga]Ga-DOTATOC	17 patients (104 lesions)	Lesion-based detection was higher with FDG than DOTATOC in radioiodine-positive (28/31 vs. 25/31) and radioiodine-negative subgroups (70/73 vs. 26/73; *p* < 0.01).	SSTR PET may provide additional biological information in refractory or recurrent DTC.
**NSCLC**	Blum et al. [59]	SPECT	[^99m^Tc]Tc-depreotide	114 patients	For solitary pulmonary nodule characterization, it showed 96.6% sensitivity (85/88) and 73.1% specificity (19/26), using histology as the reference standard.	Demonstrated high sensitivity for characterization of solitary pulmonary nodules.
	Kahn et al. [60]	SPECT	[^99m^Tc]Tc-depreotide	166 enrolled (157 with evaluable lung lesions)	Patient-based sensitivity/specificity for malignancy were 94%/51% with depreotide versus 96%/71% with FDG PET. Correct staging was achieved in 63/139 (45%) versus 76/139 patients (55%), respectively.	^99m^Tc-depreotide may detect primary tumors, but FDG remains superior for staging.
	Wang et al. [61]	SPECT	[^99m^Tc]Tc-octreotide	44 patients (25 NSCLC, 6 SCLC, and 13 benign lung lesions)	Patient-based sensitivity was 100% (31/31) for primary tumors with both tracers, but 35% (7/20) for hilar/mediastinal nodal involvement with octreotide versus 100% (20/20) with FDG.	Octreotide showed sensitivity comparable to FDG DHC for primary tumors but lower sensitivity for hilar/mediastinal nodal involvement.
	Rezaeetalab et al. [62]	SPECT	[^99m^Tc]Tc-HYNIC-TATE	21 patients with solitary pulmonary nodules (7 malignant; 14 benign)	For solitary pulmonary nodule characterization, [^99m^Tc]Tc-HYNIC-TATE showed patient-based sensitivity of 100% (7/7), specificity of 79% (11/14), and accuracy of 86% (18/21).	Showed comparable diagnostic performance for SPN evaluation.
	Wang et al. [63]	SPECT	[^99m^Tc]Tc-octreotide	29 patients (16 NSCLC, 2 SCLC, and 11 benign lung lesions)	Patient-based specificity for pulmonary nodule characterization was 72.7% (8/11) with octreotide SPECT/CT and 81.8% (9/11) when combined with diagnostic CT, versus 63.6% (7/11) with diagnostic CT alone.	Combined diagnostic CT and SSTR SPECT/CT helped differentiate benign from malignant nodules.
	Dimitrakopoulou-Strauss et al. [64]	Dynamic PET	[^68^Ga]Ga-DOTATOC and [^18^F]F-FDG	9 patients	Mean DOTATOC SUV about 2.0; mean FDG SUV 5.7; FDG-positive metastases were DOTATOC-negative	SSTR PET showed mild-to-moderate uptake and may lose sensitivity during tumor progression.
	Walker et al. [65]	PET/CT	[^68^Ga] Ga-DOTATATE and [^18^F]F-FDG	30 patients (31 pulmonary lesions) (14 NSCLC, 1 metastatic carcinoma, and 16 benign nodules)	For lesion-based differentiation of benign and malignant pulmonary lesions, DOTATATE showed higher specificity (94% vs. 81%) but lower sensitivity (73% vs. 93%) than FDG. SSTR2A staining localized to tumor stroma rather than tumor cells.	DOTATATE had higher specificity but lower sensitivity; uptake correlated with stromal rather than tumor-cell SSTR2 expression.
Breast cancer	van Eijck et al. [11]	Scintigraphy	[^111^In]In-pentetreotide	52 patients	Nonpalpable metastatic axillary lymph nodes were detected in 4/13 patients with histologically proven axillary metastases.	SSTR scintigraphy can visualize primary tumors but has limited performance for axillary nodal metastases.
	Vural et al. [71]	Scintigraphy	[^111^In]In-octreotide and ^201^Tl	21 patients	[^111^In]In-octreotide detected 16 of the 17 breast primary cancers (94%) while ^201^Tl detected 13 (76%).	Early evidence for SSTR-based imaging in suspected breast cancer.
	Van den Bossche et al. [72]	Planar scintigraphy + SPECT	[^99m^Tc]Tc-depreotide	20 enrolled (18 evaluable)	baseline [^99m^Tc]Tc-depreotide scintigraphy for endocrine responsiveness: PPV 73% (8/11), NPV 100% (7/7); baseline + 3-week dynamic change predicted response with 100% accuracy; responders: decreased uptake; nonresponders: increased uptake (*p* = 0.017)	[^99m^Tc]Tc-depreotide scintigraphy may allow early separation of responders and nonresponders as early as 3 weeks after initiating endocrine therapy.
	Albérini et al. [73]	Scintigraphy	[^111^In]In-pentetreotide	13 patients (11 malignant primary tumors and 6 malignant axillary nodes)	Primary tumors: 4/11 (36.4%) visible (6/11 expressed SSTR in vitro); axillary nodes: 2/6 (33.3%) visible (4/6 expressed SSTR)	^111^In-pentetreotide lacks sufficient sensitivity for detecting axillary lymph node metastases in breast cancer.
	Wang et al. [74]	Scintigraphy	[^99m^Tc]Tc-octreotide acetate	55 patients (37 with malignant and 18 with benign breast lesions)	For primary breast lesion assessment, octreotide scintigraphy had reported sensitivity/specificity of 91.8% (34/37) /22.2% (4/18). Uptake correlated with SSTR2 mRNA expression (r = 0.73; *p* < 0.01).	High sensitivity but low specificity for primary breast lesion detection; detection of axillary metastases was limited by nonspecific uptake in normal breast tissue and low tumor-to-background contrast.
	Nguyen et al. [75]	PET/CT	[^68^Ga]Ga-DOTATATE and [^18^F]F-FDG	10 patients Metastatic ER-positive/PR-positive/HER2-negative breast cancer	8/10 patients had identifiable lesions with both tracers; some bone metastases were DOTATATE-positive/FDG-negative, while most visceral lesions were FDG-positive with minimal DOTATATE uptake.	Demonstrates inter-lesional heterogeneity and possible value in selected bone-dominant disease.
	Chandekar et al. [76]	PET/CT	[^68^Ga]Ga-DOTATATE and [^18^F]F-FDG	30 patients	Patient-level detection rates were 100% for FDG and 96.7% for DOTATATE. FDG detected more visceral/other metastatic lesions (235 vs. 128; *p* = 0.003). DOTATATE yielded a lower M-stage in 10% of patients; 3/30 had patient-level Krenning scores ≥3.	Only a small subgroup may be eligible for SSTR-targeted theranostics.
	Ulaner et al. [77]	PET/CT	[^68^Ga]Ga-DOTATATE	30 (imaging); 1 (treated)	9/30 (30%) had Krenning scores 3–4; in proof-of-concept, 1 of 9 received ^225^Ac-DOTATATE and achieved near-complete response	Dotatate PET/CT can identify candidates; therapeutic experience limited to single proof-of-concept case requiring validation.
Lymphoma	Ivancević et al. [83]	Scintigraphy	[^111^In]In-pentetreotide	35 patients (19 undergoing initial staging; 16 undergoing restaging)	Overall patient-based sensitivity was 88%; lesion-based sensitivities were 57%, 35%, and 43% for HD, low-grade NHL, and high-grade NHL, respectively. Understaging occurred in 22/35 patients.	Limited value for lymphoma staging.
	Apostolopoulos et al. [84]	SPECT	[^99m^Tc]Tc-depreotide vs. ^67^Ga-citrate	47 HL and 59 NHL patients	^99m^Tc-depreotide detected more lesions than ^67^Ga in HL and indolent NHL, upstaged 32% of early-stage HL, but showed low sensitivity for abdominal (22.7%) and bone marrow (36.4%) involvement.	Limited value for initial staging; may be useful post-therapy for recurrence detection in indolent lymphomas.
	Raderer et al. [85]	Scintigraphy	[^111^In]In-pentetreotide	30 patients	^111^In-octreotide scintigraphy was positive in all 24 primary extragastric MALT lymphomas, detected persistence/relapse earlier than conventional imaging, but negative in all post-gastric dissemination cases.	Potential role in staging and follow-up of MALT lymphoma.
	Li et al. [86]	Scintigraphy	^67^Ga-citrate, [^111^In]In-DOTA-TOCT, [^111^In]In-DOTA-LAN	18 patients	^67^Ga detected 63% of patients and 58% of involved sites; SSTR tracers detected 56%, 55% of lesions, respectively.	SSTR-targeted imaging is feasible in MALT lymphoma but detection is moderate.
Melanoma	Valencak et al. [88]	Scintigraphy	[^111^In]In-DOTA-lanreotide and [^111^In]In-DOTA-Tyr3-octreotide	23 patients Stage IV melanoma (61 lesions and 23 lesions)	Lesion-based detection rates were 62% (38/61) with [^111^In]In-DOTA-lanreotide and 43% (10/23) with [^111^In]In-DOTA-Tyr^3^-octreotide.	Scintigraphy can visualize metastatic melanoma lesions, but has no direct therapeutic antiproliferative effect.
	Brogsitter et al. [89]	PET/CT	[^68^Ga]Ga-DOTATOC and [^18^F]F-FDG	18 patients	61% of patients had DOTATOC-positive metastases, but only 22% of FDG-avid lesions were DOTATOC-positive; DOTATOC SUVmax 3.1 vs. FDG SUVmax 28.2.	SSTR PET has limited lesion detection and is unlikely to provide effective therapeutic targeting in metastatic melanoma.

Note: SPECT, single photon emission computed tomography; PET, positron emission tomography; CT, computed tomography; MRI, magnetic resonance imaging; PPV, positive predictive value; NPV, negative predictive value; SUVmax, maximum standardized uptake value; L/B ratio, lesion-to-background ratio; T/NT, tumor-to-normal tissue ratio; SPN, solitary pulmonary nodule; ER, estrogen receptor; PR, progesterone receptor; HER2, human epidermal growth factor receptor 2; EBV, Epstein–Barr virus; DTC, differentiated thyroid carcinoma; RAI, radioiodine; MALT, mucosa-associated lymphoid tissue; HD, Hodgkin lymphoma; NHL, non-Hodgkin lymphoma; ^67^Ga, gallium-67; ^18^F-FDG, fluorine-18 fluorodeoxyglucose; BBB, blood–brain barrier. Some pulmonary studies included benign lesions, SCLC [61,63], diagnostic results are not NSCLC-specific.

**Table 4:** PRRT evidence in non-neuroendocrine tumors.

Tumor Type	Study	Study Design	Patients (*n*)	Radio-pharmaceutical	Response	Toxicity	Key Findings
Meningioma	Kurz et al. [24]	Phase II, Prospective	14	[^177^Lu]Lu-DOTATATE	PFS-6: 50% (7/14); SD was the best radiographic response in all 7 patients	No grade 3–4 renal/hepatic	PFS-6 endpoint was met; >25% reduction in [^68^Ga]Ga-DOTATATE uptake was observed in 5 meningiomas from 2 patients.
	Amerein et al. [25]	Retrospective	13	[^177^Lu]Lu-HA-DOTATATE	10/13 DC (1 CR, 1 PR, 8 SD)	Grade 1–2 hematologic	Intra-arterial PRRT was feasible and showed encouraging disease control with predominantly grade 1–2 hematologic toxicity.
	LUMEN-1 (NCT06326190)	Phase II RCT (ongoing)	136 (planned)	[^177^Lu]Lu-DOTATATE	Pending (PFS at 2 yr)	Pending	Ongoing randomized phase II trial evaluating [^177^Lu]Lu-DOTATATE versus standard of care in recurrent meningioma.
Glioma	Schumacher et al. [32]	Pilot	10	[^90^Y]Y-DOTATOC	2 CR, 3 PR, 5 SD	Well tolerated	Locoregional PRRT was feasible, with reported responses lasting 13–45 months; one anaplastic astrocytoma became resectable.
	Heute et al. [33]	Pilot	3	[^90^Y]Y-DOTATOC	1 CR, 2 PR	Minor	Via catheter feasible; correlated with clinical/ quality of life improvement
NPC	Chen et al. [40]	Preclinical + *n* = 1	1	[^177^Lu]Lu-LNC1010	Favorable (case report)	Negligible	Preclinical studies showed higher uptake, retention, and antitumor activity than DOTATATE; the single-patient experience provides preliminary proof-of-concept.
DTC	Roll et al. [50]	Retrospective	5	[^177^Lu]Lu-DOTATATE	1 PR, 3 PD, 1 discordant	NR	Heterogeneous responses; only 1/5 PR despite good uptake
	Teunissen et al. [51]	Retrospective	5 (3 HCTC, 1 PTC, 1 FTC)	[^177^Lu]Lu-DOTATATE	1 PR, 1 minor, 2 SD, 1 PD	NR	Effective in DTC, esp. HCTC; uptake ratio ≥2.4 correlated with response.
	Czepczyński et al. [52]	Retrospective	11 (6 completed)	[^90^Y]Y-DOTATOC	1 PR, 2 SD, 3 PD	Minor, transient hematologic	Median OS was 21 months; disease control was observed in a subset of patients with RAI-refractory DTC.
	Versari et al. [53]	Prospective	41 screened; 13 eligible; 11 treated	[^90^Y]Y-DOTATOC	2 PR, 5 SD (7/11 DC; 3.5–11.5 mo)	Nausea, asthenia, transient hematologic; 1 permanent renal	SSTR-positive functional volume was associated with treatment response (*p* = 0.001).
	Lee & Kim [54]	Meta-analysis	67 DTC	Lu- or Y-based PRRT	Overall: ORR 15.61%, DCR 53.95%; [^177^Lu]Lu: ORR 24.52%, DCR 67.26%; [^90^Y]Y: ORR 13.82%, DCR 50.86%	Serious adverse events: 2.82% (95% CI, 0.03–17.61%)	PRRT showed modest response rates in DTC; pooled ORR and DCR were numerically higher with ^177^Lu-based than with ^90^Y-based PRRT.
BC	Ulaner et al. [77]	Phase II imaging + *n* = 1	30 imaging; 1 treated	[^225^Ac]Ac-DOTATATE	Near-CR (*n* = 1)	Dry mouth, transient cytopenia	9/30 (30%) had Krenning scores 3–4; treatment experience was limited to a single proof-of-concept case.

Note: NPC, nasopharyngeal carcinoma; DTC, differentiated thyroid carcinoma; HCTC, Hurthle cell thyroid carcinoma; PTC, papillary thyroid carcinoma; FTC, follicular thyroid carcinoma; BC, breast cancer; PFS, progression-free survival; SD, stable disease; CR, complete response; PR, partial response; DC, disease control; OS, overall survival; PD, progressive disease; ORR, objective response rate; DCR, disease control rate; IV, intravenous; RCT, randomized controlled trial; SOC, standard of care; NR, not reported; RAI, radioiodine; FV, functional volume; ER, estrogen receptor; SSTR, somatostatin receptor. The meta-analysis by Lee and Kim [54] included 98 patients with medullary thyroid carcinoma and 67 patients with DTC. Because this table is restricted to non-neuroendocrine tumors, only the DTC-specific results are presented; medullary thyroid carcinoma outcomes were excluded and were not used to derive the values shown.

## Data Availability

No new data were created or analyzed in this study. Data sharing is not applicable to this article.

## Abbreviations

CAF	cancer-associated fibroblast
CT	computed tomography
DCR	disease control rate
DTC	differentiated thyroid carcinoma
EBV	Epstein–Barr virus
ER	estrogen receptor
FDG	fluorodeoxyglucose
IHC	immunohistochemistry
K-NPC	keratinizing nasopharyngeal carcinoma
MALT	mucosa-associated lymphoid tissue
MRI	magnetic resonance imaging
NET	neuroendocrine tumor
NK-NPC	non-keratinizing nasopharyngeal carcinoma
NPC	nasopharyngeal carcinoma
NSCLC	non-small cell lung cancer
ORR	objective response rate
PET	positron emission tomography
PFS	progression-free survival
PRRT	peptide receptor radionuclide therapy
RT-PCR	reverse transcription polymerase chain reaction
SCLC	small cell lung cancer
SPECT	single photon emission computed tomography
SSTR	somatostatin receptor
TAM	tumor-associated macrophage
